# Identification of immune genes and proteins involved in the response of bovine mammary tissue to *Staphylococcus aureus *infection

**DOI:** 10.1186/1746-6148-4-18

**Published:** 2008-06-02

**Authors:** Ylva C Strandberg Lutzow, Laurelea Donaldson, Christian P Gray, Tony Vuocolo, Roger D Pearson, Antonio Reverter, Keren A Byrne, Paul A Sheehy, Ross Windon, Ross L Tellam

**Affiliations:** 1 Co-operative Research Centre for Innovative Dairy Products, Australia; 2 CSIRO Livestock Industries, Queensland Bioscience Precinct, 306 Carmody Rd, St Lucia, QLD 4067, Australia; 3 Centre for Advanced Technologies in Animal Genetics and Reproduction (REPROGEN), Faculty of Veterinary Science, University of Sydney, Sydney, 2006, NSW, Australia; 4 School of Veterinary Science, The University of Queensland, Brisbane, 4072, Australia; 5 CSIRO Livestock industries, FD McMaster laboratory, Chiswick, Armidale, NSW, 2350, Australia

## Abstract

**Background:**

Mastitis in dairy cattle results from infection of mammary tissue by a range of micro-organisms but principally coliform bacteria and Gram positive bacteria such as *Staphylococcus aureus*. The former species are often acquired by environmental contamination while *S. aureus *is particularly problematic due to its resistance to antibiotic treatments and ability to reside within mammary tissue in a chronic, subclinical state. The transcriptional responses within bovine mammary epithelial tissue subjected to intramammary challenge with *S. aureus *are poorly characterised, particularly at the earliest stages of infection. Moreover, the effect of infection on the presence of bioactive innate immune proteins in milk is also unclear. The nature of these responses may determine the susceptibility of the tissue and its ability to resolve the infection.

**Results:**

Transcriptional profiling was employed to measure changes in gene expression occurring in bovine mammary tissues sampled from three dairy cows after brief and graded intramammary challenges with *S. aureus*. These limited challenges had no significant effect on the expression pattern of the gene encoding β-casein but caused coordinated up-regulation of a number of cytokines and chemokines involved in pro-inflammatory responses. In addition, the enhanced expression of two genes, S100 calcium-binding protein A12 (*S100A12*) and Pentraxin-3 (*PTX3*) corresponded with significantly increased levels of their proteins in milk from infected udders. Both genes were shown to be expressed by mammary epithelial cells grown in culture after stimulation with lipopolysaccharide. There was also a strong correlation between somatic cell count, a widely used measure of mastitis, and the level of S100A12 in milk from a herd of dairy cows. Recombinant S100A12 inhibited growth of *Escherichia coli *in vitro and recombinant PTX3 bound to *E. coli *as well as C1q, a subunit of the first component of the complement cascade.

**Conclusion:**

The transcriptional responses in infected bovine mammary tissue, even at low doses of bacteria and short periods of infection, probably reflect the combined contributions of gene expression changes resulting from the activation of mammary epithelial cells and infiltrating immune cells. The secretion of a number of proinflammatory cytokines and chemokines from mammary epithelial cells stimulated by the bacteria serves to trigger the recruitment and activation of neutrophils in mammary tissue. The presence of S100A12 and PTX3 in milk from infected udder quarters may increase the anti-bacterial properties of milk thereby helping to resolve the mammary tissue infection as well as potentially contributing to the maturation of the newborn calf epithelium and establishment of the newborn gut microbial population.

## Background

Mastitis in dairy cattle continues to be an economically important disease. Despite extensive management practices including antibiotic treatments, mastitis is estimated to cause annual losses in excess of two billion dollars per annum in the United States alone [1,2]. These losses arise from reduction in milk yield, waste of milk unfit for consumption, antibiotic use and premature culling. The disease can be caused by a wide range of Gram negative coliform and Gram positive bacteria, as well as yeast, fungi and mycoplasma. However, contagious mastitis, which is transmitted from cow to cow during milking, is principally caused by *Staphylococcus aureus *[3]. Unlike many other major mastitis-causing pathogens, which are generally associated with acute infections acquired from environmental reservoirs of bacteria, *S. aureus *causes chronic subclinical infections. Control is complicated by a high prevalence of resistance to antibiotics [4]. The ability of this bacterium to live within some cells and encapsulated nodules in mammary tissue may contribute to its resistance to antibiotic treatments [5-7].

The innate immune system in mammals is one of the first lines of host defense to infection as it has the capacity to immediately recognise and respond to the earliest signs of infection. Both acute and chronic mastitis conditions are associated with a dramatic increase in somatic cell count (SCC) in milk, with neutrophils being the predominant leukocyte found in both the infected mammary quarter and milk [8,9]. Neutrophils are essential cellular components of the innate immune system [10] and their accumulation and activation at the site of infection requires local induction of a panel of innate immune molecules, including various chemokines and cytokines [11]. Early innate immune responses also occur in mammary epithelial cells and these responses underpin subsequent neutrophil infiltration into mammary tissue, the activation of these innate immune cells and increases in milk SCC [12-14].

Several studies have examined gene expression in circulating leukocytes close to parturition, a time when cows are more susceptible to mastitis infection, as well as during acute mastitis infections [10,15,16]. These studies and others using mammary tissue demonstrated that expression of a number of genes involved in immune cell activation, chemotaxis and apoptosis was altered [11,17,18]. Moreover, these gene expression changes have often been shown to translate into corresponding protein expression alterations thereby demonstrating that transcriptional mechanisms primarily control the innate immune response [19-21]. The majority of these studies used infection models that resulted in very strong mammary tissue responses, or measured the responses relatively late after experimental inoculation or during acute naturally acquired clinical mastitis when significant necrotic damage may have been evident.

Recent studies have begun to elucidate the direct role of mammary epithelial cells (MEC) in the stimulation of the innate immune response [12,14,22]. These studies indicate that MEC respond robustly and rapidly to challenge by either low levels of bacteria or bacterial cell wall components with marked changes in expression of pro-inflammatory cytokines and chemokines [23]. This response probably underpins the subsequent infiltration of mammary tissues by immune cells, particularly neutrophils [8,9]. A broader and more comprehensive examination of gene expression changes produced in mammary epithelial cells and infiltrating immune cells in response to mammary tissue infection with *S. aureus *may identify the network of gene expression changes that coordinates the early innate immune responses of this tissue. Of particular interest are the local mammary tissue responses after initial infection with low numbers of bacteria before the onset of widespread tissue damage.

Microarray technology has become an important tool to address complex biological questions by simultaneously measuring the expression of thousands of genes within a tissue. Identification of patterns of gene expression can provide insight into the biological functions of their encoded proteins and the networks of protein interactions governing responses of cells to extracellular stimuli. One aim of the current study was to identify the network of genes that becomes activated in mammary tissue in response to a brief and graded intramammary challenge with *S. aureus*. The identification of these poorly characterised genes and in-depth characterisation of their corresponding proteins may be important for the development of novel strategies to enhance resistance of cows to mastitis and the development of novel antibacterial therapeutic agents. The consequences of these responses in terms of potential secretion of bioactive innate immune proteins into milk may also have relevance to the suckling calf. Thus, a second aim is to characterise the biological activities of innate immune proteins that may be present in milk from infected udder quarters.

## Results

### S. aureus challenge of bovine mammary tissue

To ensure that the dairy cows used in the experiment were free from bacterial infection, milk samples were taken from each udder quarter 24 h and 48 h prior to intramammary *S. aureus *infusions and tested for bacterial growth. All quarters from two cows and three quarters from a third cow showed no bacterial growth. Milk from one quarter of one cow (1419) showed low level growth of *S. aureus *24 h prior to the commencement of the experiment, presumably from a mild field mastitis infection. Consequently, this quarter was excluded from the experiment. One quarter of each cow was treated with pyrogen-free PBS and this quarter acted as the intra-animal control for subsequent analyses. Each cow received different graded doses of *S. aureus *(strain JG80) by intramammary infusion, ranging from 5 × 10^2 ^to 1 × 10^6 ^bacteria (Table 1).

**Table 1:** Dose of *S. aureus *(bacteria/5 ml) given by intramammary infusion into each udder quarter

**Cow**	**Left Fore**	**Right Fore**	**Left Hind**	**Right Hind**
1490	PBS	5 × 10^2^	1 × 10^4^	1 × 10^5^
1592	1 × 10^4^	PBS	1 × 10^5^	1 × 10^6^
1419	PBS	nil^1^	1 × 10^5^	1 × 10^6^

^1 ^This quarter was not used

Milk samples were also taken from each quarter 16 h after *S. aureus *experimental infusion and tested for bacterial growth. The bacterial culture was identified and tested for hemolysis, catalase and coagulase activities (Table 2). All milk samples from the quarters experimentally infused with *S. aureus *contained bacteria with positive catalase and coagulase activities and at least partial hemolysis activity on blood agar plates. The bacteria in all of these samples were identified as *S. aureus*. The milk samples from quarters infused with pyrogen-free PBS had no *S. aureus *growth, which was consistent with the view that each quarter within an udder is a semi-autonomous physiological unit and can be considered an independent experimental unit during this relatively brief experimental bacterial challenge [24-26].

**Table 2:** Pathology of milk samples 16 h post-infusion

**Cow**	**Test**	**Left Fore**	**Right Fore**	**Left Hind**	**Right Hind**
**#1490**	Dose^1^	**PBS **	5 × 10^2^	1 × 10^4^	1 × 10^5^
	Growth^2^	Minor^8^	+	+	+
	Gramstain^3^	-	+	+	+
	Hemolysis^4^	-	partial	partial	+
	Catalase^5^	-	+	+	+
	Coagulase^6^	-	+	+	+
	Isolate^7^	n.d.	*S. aureus*	*S. aureus*	*S. aureus*

**#1592**	Dose	1 × 10^4^	**PBS **	1 × 10^5^	1 × 10^6^
	Growth	+	none	+	+
	Gramstain	+	-	+	+
	Hemolysis	partial	-	partial	partial
	Catalase	+	-	+	+
	Coagulase	+	-	+	+
	Isolate	*S. aureus*	none	*S. aureus*	*S. aureus*

**#1419**	Dose	**PBS **	Nil	1 × 10^5^	1 × 10^6^
	Growth	none	+	+	+
	Gramstain	-	+	+	+
	Hemolysis	-	partial	partial	partial
	Catalase	-	+	+	+
	Coagulase	-	+	+	+
	Isolate	none	*S. aureus*	*S. aureus*	*S. aureus*

^1 ^Bacterial dose

^2 ^Bacterial growth (+/-)

^3 ^Gram positive or Gram negative

^4 ^Hemolysis; hemolytic on blood agar (+, partial, -)

^5 ^Catalase (+/-)

^6^Coagulase (+/-)

^7^Bacterial species identified

^8 ^Minor bacterial growth was observed but this was not *S. aureus*.

**Figure 1:**
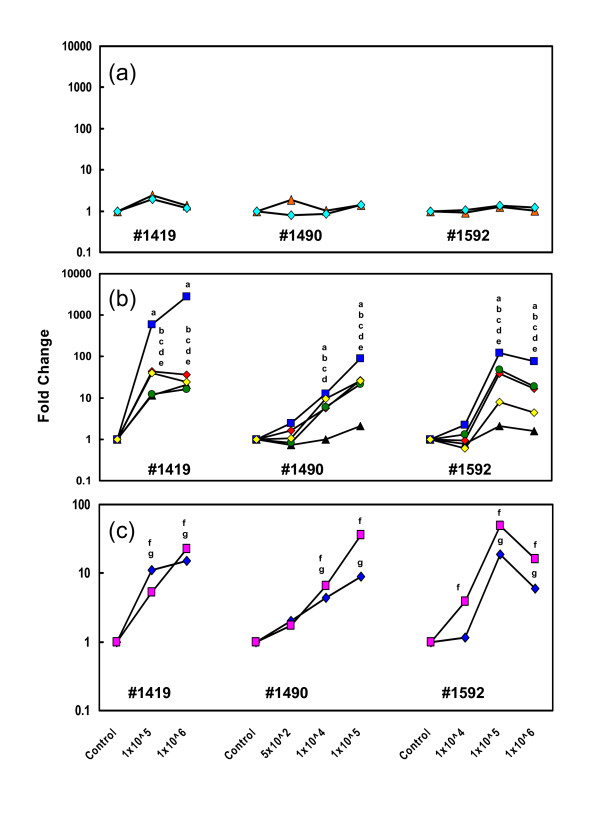
**Relative expression of selected genes in mammary tissue challenged with *S. aureus***. The relative gene expression levels of selected genes were measured by qRT-PCR. Samples were obtained from mammary tissues of three Holstein Friesian cows at peak lactation 16 hours following intramammary infusion with increasing doses of *S. aureus *into the separate udder quarters. Data for each gene are expressed as relative fold changes compared to expression in the intra-animal control tissue for each animal. The genes tested included: (a) α-S1-casein (*CSN1S1*) (turquoise diamond) and β-casein (*CSN2*) (red triangle); (b) Interleukin 8 (*IL-8*^a^) (blue square), Interleukin 1β (*IL-1β*^b^) (red diamond), Tumor necrosis factor α (*TNFα*^c^) (yellow diamond), Interleukin 6 (*IL-6*^d^) (green circle) and CD14 antigen (*CD14*^e^) (black triangle); (c) S100 calcium-binding protein A12 (*S100A12*^f^) (blue diamond) and Pentraxin-3 (*PTX3*^g^) (pink square). Expression data for each gene were analysed by ANOVA. The superscript letters are specific for each gene tested and signify significant differences (P < 0.01) between a specific infection dose and the corresponding intramammary control for each cow.

Quantitative RT-PCR (qRT-PCR) was used to measure the expression patterns of selected genes in mammary tissue samples taken from each animal 16 h after the *S. aureus *infusion (Fig. 1). There were no significant changes in the expression levels of *α-S1-casein *(*CSN1S1*) or *β-casein *(*CSN2*) as a function of bacterial dose compared with each intra-animal control sample (P > 0.05) for all three cows (Fig. 1(a)). Therefore, the relatively low doses of *S. aureus *in combination with the relatively brief infection period did not affect the expression of these milk protein genes in any quarter. This indicates that the experimental model was a good representation of the early stages of infection before there was significant tissue damage leading to the suppression of normal physiological functions.

The expression patterns of the pro-inflammatory cytokines tumor necrosis factor alpha (*TNFα*), interleukin 1 beta (*IL-1β*), interleukin 8 (*IL-8*), interleukin 6 (*IL-6*) and CD14 antigen (*CD14*), a pivotal cell surface protein mediating detection of bacterial cell wall components, were measured as representatives of early response innate immune genes that are typically up-regulated by bacterial infections of mammary tissue [12,14,27]. For each of these genes, and within each cow, there were strong and significant responses to the highest bacterial doses compared with each intra-animal control (P < 0.01) (Fig. 1(b)). Moreover, the responses were dose-dependent, or at least partially so. However, the magnitudes of the fold-changes in gene expression were variable across the three cows for each gene. For example, at a dose of 1 × 10^5 ^bacteria, the fold changes for *TNFα *were 39.8 (cow 1419), 26.2 (cow 1490) and 7.8 (cow 1592) (all P < 0.01). Furthermore, the responses of samples from cow 1419 for all of the measured pro-inflammatory cytokines were more pronounced than the responses in the two other cows. For *IL-8 *the fold changes were much greater compared with *TNFα *and also more pronounced in cow 1419. The fold changes for *IL-8 *were 603 and 2835 at the bacterial doses of 1 × 10^5 ^and 1 × 10^6^, respectively for cow 1419, while corresponding figures for cow 1592 were 122 and 75 fold (all P < 0.01).

### Microarray analyses of mammary tissue challenged with different doses of S. aureus

**Figure 2:**
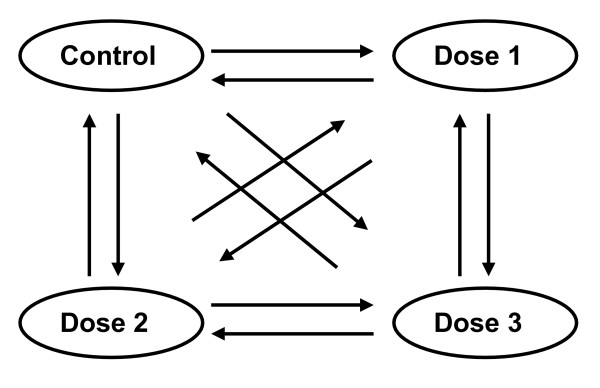
**Microarray experimental design**. Gene expression in mammary tissue samples from each cow was analysed by microarray hybridisation using an 'all-pairs design'. The figure is a schematic representation of the microarray design used for each cow (except cow 1419 which only had 2 intramammary bacterial infusions), where each arrow represents one microarray slide with the direction of the arrow indicating the cDNA labelling from Cy5 to Cy3-labelled cDNA.

**Figure 3:**
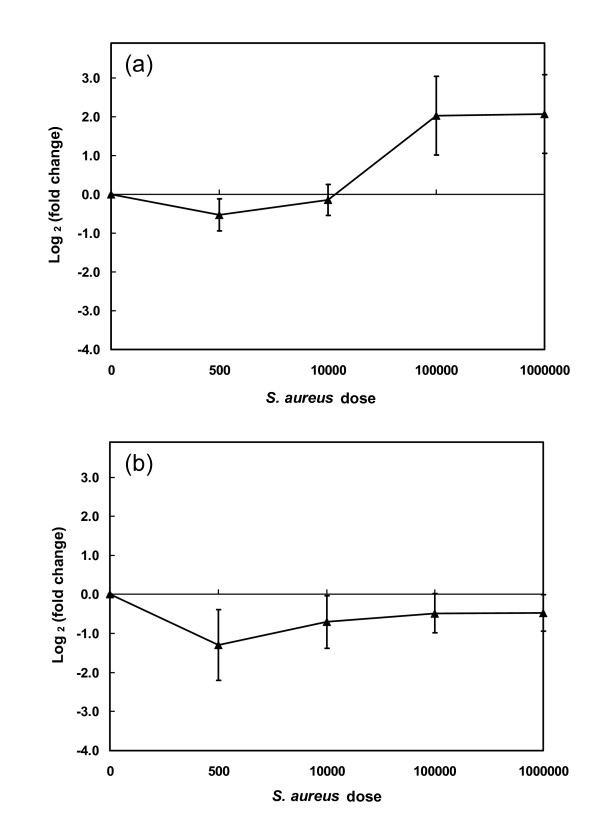
**Clusters of differentially expressed genes in response to intramammary infusion of *S. aureus***. Microarrays were performed on tissue from each mammary quarter with data compared to the intramammary control for each cow to identify differentially expressed transcripts. The figure shows the mean gene expression cluster profiles for the significantly differentially expressed elements that were either up-regulated (a) or down-regulated (b) relative to the control in response to intramammary infusion of *S. aureus*. The clusters only contain significantly differentially expressed elements that were expressed in all samples and showed at least 2 fold change relative to the control in at least one sample. RNA samples were obtained from mammary tissue of three Holstein Friesian cows at peak lactation that had been infused with pyrogen-free PBS (dose 0, *n *= 3) in one quarter (intra-animal controls) and in other quarters with *S. aureus *at dose rates of 500 (*n *= 1), 10,000 (*n *= 2), 100,000 (*n *= 3) or 1,000,000 (*n *= 2) bacteria. Data are expressed as log_2 _(fold change) ± 1 S.D. for the centroid of the cluster.

We used the Bovine Innate Immune Microarray [28] to identify a more complete repertoire of differentially expressed transcripts in bovine udder tissues from the three dairy cows challenged by intramammary infusion with *S. aureus*. It was clear from the cow to cow variations in the initial gene expression data that each cow required independent assessment (Fig. 1(b)). Consequently, an 'All-Pairs' experimental design was used for each cow i.e. each sample taken from one cow was tested against all other samples from the same cow with each comparison also involving dye swaps (Fig. 2). In this manner, every sample was measured a total of six times, which allowed identification of differentially expressed genes with respect to the intra-animal control with a False Discovery Rate (FDR) adjusted P < 0.05 [29]. Differentially expressed elements were independently identified for each cow. In a conservative extension of this analysis, a subset of the significantly differentially expressed elements was selected that had: (i) expression data in all test samples and; (ii) showed at least 2-fold change in expression in any of the test samples compared to the control sample. The signal for each of these 183 differentially expressed elements was then averaged in all cows receiving the same dose. In this way, gene expression values were obtained for these genes from the test quarters infused with *S. aureus *at dose rates of 500 (*n *= 1), 10,000 (*n *= 2), 100,000 (*n *= 3) or 1,000,000 (*n *= 2) bacteria relative to the control quarters. The expression data for these 183 elements were clustered using a K-means algorithm into 9 clusters using default parameters [30]. The gene redundancy on the microarray revealed overlap between several of the clusters. Consequently, these clusters were manually collapsed into two primary clusters in which the significantly differentially expressed elements were either reduced (repressed) or increased (induced) in response to *S. aureus *infection (Fig. 3). Each cluster is represented by the centroid of gene expression for all elements in the cluster. The 128 elements that showed increased expression in response to *S. aureus *challenge are shown in Figure 3(a), while the 55 elements with decreased gene expression in response to *S. aureus *are presented in Figure 3(b).

The cluster containing elements that were up-regulated in response to infection showed increased gene expression only as the number of infused bacteria reached a threshold dose i.e. the centroid for this cluster showed little response at a dose up to 10,000 bacteria but a strong and sustained positive response at higher doses. As expected from the data, there was substantial variation in the responses of individual genes at the higher doses (cf Fig. 1). The characteristics of this cluster are exemplified by the responses of genes such as *IL-6, IL-1β *and *IL-8 *(Fig. 1(b)). The cluster containing down-regulated genes showed suppression of gene expression compared to the controls at all bacterial doses, although these effects were not as marked.

A number of the differentially expressed elements identified by this analysis were anonymous on the microarray and consequently inserts from the corresponding cDNA clones were sequenced and annotated. This process revealed that the 128 up-regulated elements represented 56 unique transcripts (Table 3) while the 55 down-regulated elements represented 42 unique transcripts (Table 4). This reflected the redundancy of elements on the microarray and the technical replication of the data. Due to the biased representation of innate immune genes on the microarray, a Gene Ontology (GO) analysis was not performed to identify enriched GO terms. For convenience of presentation and discussion however, the differentially expressed genes were placed in several functional categories, which were derived by direct examination of the scientific literature (Tables 3 and 4).

**Table 3:** Significantly up-regulated genes in mammary tissue challenged with *S. aureus*.

***Function***	***Gene symbol***	***Genbank accession***	***Genbank description***
***Chemokine, cytokine and intercellular signalling***			
	*CCL11*	NM_002986	Homo sapiens chemokine (C-C motif) ligand 11 (CCL11), mRNA
	*CCL2*	NM_174006	Bos taurus chemokine (C-C motif) ligand 2 (CCL2), mRNA.
	*CCL20*	NM_004591	Homo sapiens chemokine (C-C motif) ligand 20 (CCL20), mRNA
	*CCL3L1*	NM_021006	Homo sapiens chemokine (C-C motif) ligand 3-like 1 (CCL3L1), mRNA
	*CCL4L1*	AY575854	Homo sapiens similar to cytokine (LOC388372), mRNA
	*CSF3*	NM_172219	Homo sapiens colony stimulating factor 3 (granulocyte) (CSF3), transcript variant 2, mRNA
	*CXCL1*	NM_001511	Homo sapiens chemokine (C-X-C motif) ligand 1 (melanoma growth stimulating activity,
	*CXCL2*	NM_002089	Homo sapiens chemokine (C-X-C motif) ligand 2 (CXCL2), mRNA
	*CXCL10*	AB070717	Homo sapiens chemokine (C-X-C motif) ligand 10 (CXCL10), mRNA
	*IL1B*	NM_174093	Bos taurus interleukin 1, beta (IL1B), mRNA.
	*IL6*	NM_001009392	Ovis aries interleukin 6 (IL6), mRNA
	*IFNG*	NM_174086	interferon-gamma; Bos taurus interferon, gamma or immune type [interferon gamma type 2]
	*IL8*	NM_173925	Bos taurus interleukin 8 [neutrophil activating peptide 1] (IL8), mRNA.
	*LEP*	NM_173928	Bos taurus leptin [obesity] (LEP), mRNA
	*PLAUR*	NM_174423.2	Homo sapiens plasminogen activator, urokinase receptor (PLAUR), mRNA
	*PTX3*	NM_002852	Homo sapiens pentaxin-related gene, rapidly induced by IL-1 beta (PTX3), mRNA
	*S100A12*	NM_174651	Bos taurus S100 calcium binding protein A12 (calgranulin C) (S100A12), mRNA
	*S100A2*	NM_005978	Homo sapiens S100 calcium binding protein A2 (S100A2), mRNA
	*THBS1*	NM_174196	Homo sapiens thrombospondin 1 (THBS1), mRNA
			
***Cell surface receptors***			
	*CCR1*	NM_001295	Homo sapiens chemokine (C-C motif) receptor 1 (CCR1), mRNA
	*CD14*	NM_000591	Homo sapiens CD14 antigen (CD14), mRNA
	*CD40*	NM_152854	Homo sapiens tumor necrosis factor receptor superfamily, member 5 (TNFRSF5), transcript
	*CSF3R*	NM_172313	Homo sapiens colony stimulating factor 3 receptor (granulocyte) (CSF3R), transcript variant
	*IL11RA*	NM_147162	Homo sapiens interleukin 11 receptor, alpha (IL11RA), transcript variant 2, mRNA
	*SLC2A4*	NM_001042	Homo sapiens solute carrier family 2 (facilitated glucose transporter), member 4 (SLC2A4)
	*TLR2*	NM_003264	Homo sapiens toll-like receptor 2 (TLR2), mRNA
	*TLR4*	NM_138557	Homo sapiens toll-like receptor 4 (TLR4), transcript variant 4, mRNA
	*TNFRSF9*	NM_001561	Homo sapiens tumor necrosis factor receptor superfamily, member 9 (TNFRSF9), mRNA
			
***Intracellular signaling***			
	*CDKN1A*	NM_078467	Homo sapiens cyclin-dependent kinase inhibitor 1A (p21, Cip1) (CDKN1A), transcript variant 2, mRNA
	*FGD6*	AC009159	Homo sapiens FYVE, RhoGEF and PH domain containing 6 (FGD6), mRNA
	*MTMR10*	AJ567991	Homo sapiens hypothetical protein FLJ20313 (FLJ20313), mRNA
	*HCK*	NM_002110	Homo sapiens hemopoietic cell kinase (HCK), mRNA
	*MARCKS*	NM_002356	Homo sapiens myristoylated alanine-rich protein kinase C substrate (MARCKS), mRNA
	*MARCKSL1*	NM_023009	Homo sapiens MARCKS-like protein (MLP), mRNA
	*ZFYVE9*	NM_007324	Homo sapiens MAD, mothers against decapentaplegic homolog (Drosophila) interacting
			
***Apoptosis***			
	*BAT3*	NM_174196	Homo sapiens HLA-B associated transcript 3 (BAT3), transcript variant 3, mRNA
	*BCL2A1*	NP_004040	Homo sapiens BCL2-related protein A1 (BCL2A1), mRNA
	*BIRC3*	NM_182962	Homo sapiens baculoviral IAP repeat-containing 3 (BIRC3), transcript variant 2, mRNA
	*IER3*	NM_003897	Homo sapiens immediate early response 3 (IER3), transcript variant short, mRNA
			
***Transcription factor or regulator***			
	*ATF3*	NM_001674	Homo sapiens activating transcription factor 3 (ATF3), mRNA
	*BTG2*	NM_006763	Homo sapiens BTG family, member 2 (BTG2), mRNA
	*EGR1*	NM_001964	Homo sapiens early growth response 1 (EGR1), mRNA
	*FOS*	NM_005252	Homo sapiens v-fos FBJ murine osteosarcoma viral oncogene homolog (FOS), mRNA
	*JUNB*	NM_002229	Homo sapiens jun B proto-oncogene (JUNB), mRNA
	*NFKB2*	NM_002502	Homo sapiens nuclear factor of kappa light polypeptide gene enhancer in B-cells
	*NFKBIA*	NM_020529	Homo sapiens nuclear factor of kappa light polypeptide gene enhancer in B-cells inhibitor
	*NR2F6*	AF338825	Homo sapiens nuclear receptor subfamily 2, group F, member 6 (NR2F6), mRNA
			
***Assorted***			
	*ACSL6*	NM_015256	Homo sapiens acyl-CoA synthetase long-chain family member 6 (ACSL6), mRNA
	*FUT8*	NM_177501	Homo sapiens fucosyltransferase 8 (alpha (1,6) fucosyltransferase) (FUT8), transcript
	*HSPA1A*	NM_005345	Homo sapiens heat shock 70 kDa protein 1A (HSPA1A), mRNA
	*MGC21881*	XP_219130	Homo sapiens hypothetical protein LOC286286 (LOC286286), mRNA
	*NOS2A*	NM_000625	Homo sapiens nitric oxide synthase 2A (inducible, hepatocytes) (NOS2A), transcript variant
	*SOD2*	L22092	Homo sapiens superoxide dismutase 2, mitochondrial (SOD2), mRNA
	*TIMP1*	NM_003254	Homo sapiens tissue inhibitor of metalloproteinase 1 (erythroid potentiating activity, collagenase)
	*TUBA1*	NM_006000	Homo sapiens tubulin, alpha 1 (testis specific) (TUBA1), mRNA
	*WARS*	NM_174218	Homo sapiens tryptophanyl-tRNA synthetase (WARS), mRNA

**Table 4:** Significantly down-regulated genes in mammary tissue challenged with *S. aureus*.

**Function**	**Gene symbol**	**Genbank accession**	**Genbank description**
***Chemokine, cytokine and intercellular signalling***			
	*IL1A*	M37210	Bovine interleukin 1-alpha (IL-1-alpha) mRNA, complete cds.
	*NGFB*	NM_002506	Homo sapiens nerve growth factor, beta polypeptide (NGFB), mRNA
	*IL13*	AF072807	Bos taurus interleukin-13 precursor (IL-13) mRNA, partial cds
	*RETN*	NM_183362	Bos taurus resistin (RETN), mRNA
	*SFRP1*	NM_003012	Homo sapiens secreted frizzled-related protein 1 (SFRP1), mRNA
	*SPP1*	AF492837	Homo sapiens secreted phosphoprotein 1 (osteopontin, bone sialoprotein I, early T-lymphocyte activation 1) (SPP1), mRNA
			
***Cytoskeleton and extracellular matrix***			
	*CD24*	NM_013230	Homo sapiens CD24 antigen (small cell lung carcinoma cluster 4 antigen) (CD24),
	*EPB41L1*	NM_174528	Homo sapiens erythrocyte membrane protein band 4.1-like 1 (EPB41L1), transcript
	*FLNA*	NM_001456	Homo sapiens filamin A, alpha (actin binding protein 280) (FLNA), mRNA
	*ITGA3*	NM_002204	Homo sapiens integrin, alpha 3 (antigen CD49C, alpha 3 subunit of VLA-3 receptor
	*ITGB4*	NM_000213	Homo sapiens v-kit Hardy-Zuckerman 4 feline sarcoma viral oncogene homolog
	*KRT5*	BC006780	Homo sapiens keratin 5 (epidermolysis bullosa simplex, Dowling-Meara/Kobner/Weber
	*PFN2*	NM_053024	Homo sapiens profilin 2 (PFN2), transcript variant 1, mRNA
	*VCAM1*	NM_174484	Homo sapiens vascular cell adhesion molecule 1 (VCAM1), transcript variant 2, mRNA
	*VCL*	NM_014000	Homo sapiens vinculin (VCL), transcript variant meta-VCL, mRNA
	*VIM*	NM_003380	Homo sapiens vimentin (VIM), mRNA
			
***Cell surface receptors***			
	*CX3CR1*	NM_001337	Homo sapiens chemokine (C-X3-C motif) receptor 1 (CX3CR1), mRNA
	*ERBB4*	NM_005235	Homo sapiens v-erb-a erythroblastic leukemia viral oncogene homolog 4 (avian)
	*FGFR1*	NM_023106	Homo sapiens fibroblast growth factor receptor 1 (fms-related tyrosine kinase 2)
	*KIT*	NM_000222	Homo sapiens v-kit Hardy-Zuckerman 4 feline sarcoma viral oncogene homolog (KIT)
	*MET*	V00654	Homo sapiens met proto-oncogene (hepatocyte growth factor receptor) (MET), mRNA
	*PDGFRA*	NM_006206	Homo sapiens platelet-derived growth factor receptor, alpha polypeptide (PDGFRA)
	*TGFBR3*	NM_003243	Homo sapiens transforming growth factor, beta receptor III (betaglycan, 300 kDa)
			
***Intracellular signaling***			
	*PP*	M95283	Homo sapiens pyrophosphatase (inorganic) (PP), mRNA
	*PPM1J*	NM_005167	Homo sapiens protein phosphatase 1J (PP2C domain containing) (PPM1J), mRNA
	*PRKCA*	NM_174435	Homo sapiens protein kinase C, alpha (PRKCA), mRNA
	*RHOB*	NM_004040	Homo sapiens ras homolog gene family, member B (RHOB), mRNA
			
***Apoptosis***			
	*CIDEA*	NM_001279	Homo sapiens cell death-inducing DFFA-like effector a (CIDEA), transcript variant
	*CLU*	NM_173902	Bos taurus clusterin (CLU), mRNA.
	*EPHA1*	NM_005232	Homo sapiens EphA1 (EPHA1), mRNA
	*EPHA7*	NM_004440	Homo sapiens EphA7 (EPHA7), mRNA
			
***Transcription factor or regulator***			
	*DDX5*	NM_004396	Homo sapiens DEAD (Asp-Glu-Ala-Asp) box polypeptide 5 (DDX5), mRNA
	*NFATC4*	NM_004554	Homo sapiens nuclear factor of activated T-cells, cytoplasmic, calcineurin-dependent
	*TEBP*	NM_006601	Homo sapiens inactive progesterone receptor, 23 kD (TEBP), mRNA
			
***Assorted***			
	*CAMP*	NM_174825	Homo sapiens cathelicidin antimicrobial peptide (CAMP), mRNA
	*CSN1S1*	NM_181029	Homo sapiens casein alpha s1 (CSN1S1), mRNA
	*FLJ20920*	NM_025149	Homo sapiens hypothetical protein FLJ20920 (FLJ20920), mRNA
	*GATM*	NM_001482	Homo sapiens glycine amidinotransferase (L-arginine:glycine amidinotransferase
	*GPX4*	NM_002085	Homo sapiens glutathione peroxidase 4 (phospholipid hydroperoxidase) (GPX4)
	*LOC529035*	AF109167	Bos taurus immunoglobulin IgA heavy chain constant region gene, partial cds
	*18S rRNA*	AY150548	Cervus nippon 18S ribosomal RNA gene, partial sequence
	*SNX19*	AC150499	Homo sapiens similar to Sorting nexin 19 (LOC399979), mRNA

The up-regulated genes were dominated by 22 genes encoding proteins involved in intercellular signalling. Many of these were well defined pro-inflammatory cytokines and chemokines (eg. CXCL1, CXCL2, IFNγ, IL-6, IL-8 and IL-1β) as well as proteins with less defined functions but associated with inflammatory responses (eg. S100A12 and PTX3) [31,32]. Interestingly, this group of genes also included leptin (*LEP*), alluding to a link between infection and energy partitioning in the form of lipid storage and metabolism [33]. Another group (10 genes) associated with the up-regulated genes was characterised as cell surface receptors. Many of these are intimately associated with the previous functional group. For example, the Toll-like receptor pathway senses and responds to bacterial infections through the Toll-like receptor signalling pathway resulting in stimulation of synthesis of a number of pro-inflammatory cytokines and chemokines [34]. This specific pathway is represented by toll-like receptor 2 (*TLR2*), toll-like receptor 4 (*TLR4*) and CD14 [35,36]. Both TLR2 and TLR4 are membrane-bound pattern recognition receptors. In addition, the cell surface receptor group contains TNF receptor superfamily 9 (*TNFRSF9*) and CD40 antigen (*CD40*), both of which are components of the TNF receptor superfamily. The up-regulation of a group of transcription factors and transcriptional co-regulators (8 genes) may be underlying the transcriptional changes of other genes in these clusters. Many of these former genes encode proteins that are pivotal regulators of innate immune responsiveness e.g. FOS, JUN, NF-κB2, NF-κB1A [37]. A small group of genes involved in apoptosis was also identified.

The cluster containing the down-regulated genes was dominated by 10 genes encoding proteins which are components of the cytoskeleton or extracellular matrix. Thus, even over the relatively short duration of the infection, the beginning of a tissue remodelling process may have been in progress, although this did not adversely affect the transcription of milk protein genes. Other major functional groups in this cluster included apoptosis (4 genes), cell surface receptors (7 genes) and intercellular signalling proteins (6 genes). The latter group encoded a diverse group of proteins including interleukin 13 (IL-13), which normally suppresses proinflammatory cytokine production [38,39]. Resistin (RETN), another member of this group, is an adipocyte-secreted protein involved in type II diabetes and obesity and its identification provides a further link between energy metabolism and inflammatory responses in mammary tissue [40,41]. Somewhat surprisingly, *CAMP*, which encodes a cathelicidin anti-microbial peptide, was also in this down-regulated group. Although the qRT-PCR data indicated no significant change in the expression of αS1-casein (CSN1S1), the microarray data included this gene in the list of down-regulated genes. The reasons for this discrepancy are not clear but could reflect higher reproducibility of the microarray data relative to the qRT-PCR data for genes that are highly expressed.

### Validation of differentially expressed genes using qRT-PCR

All of the genes, with the exception of *CD14 and TNFα*, that were differentially expressed as defined by the initial qRT-PCR analyses (Fig. 1(b)), were also differentially expressed when measured by microarray analysis. The reasons why *CD14 *and *TNFα *were not identified in the microarray analysis but showed differential expression when measured by qRT-PCR are not clear but may relate to the conservative process used in the former analysis and the differing sensitivities of the two technologies. qRT-PCR was also used to verify the differential expression of two additional up-regulated genes, *S100A12 *(S100 calcium-binding protein A12) and *PTX3 *(Pentraxin 3)), identified by the microarray analysis (Fig. 1(c)). Consistent with the general trends in previous data (Fig. 1(b)), the expression patterns of *S100A12 *and *PTX3 *were significantly up-regulated within each cow but showed varying responses relative to each intra-animal control. At the common infection dose of 1 × 10^5 ^bacteria for cows 1419, 1490 and 1592, there was 5.2, 36.5 and 49.1 fold up-regulation of *S100A12 *expression and 11.1, 9.9 and 19 fold up-regulation of *PTX3 *expression, compared to the intra-animal controls, respectively (all P < 0.01) (Fig. 1(c)).

### Expression of S100A12 and PTX3 after stimulation of bovine mammary epithelial cells with lipopolysaccharide or lipoteichoic acid

**Figure 4:**
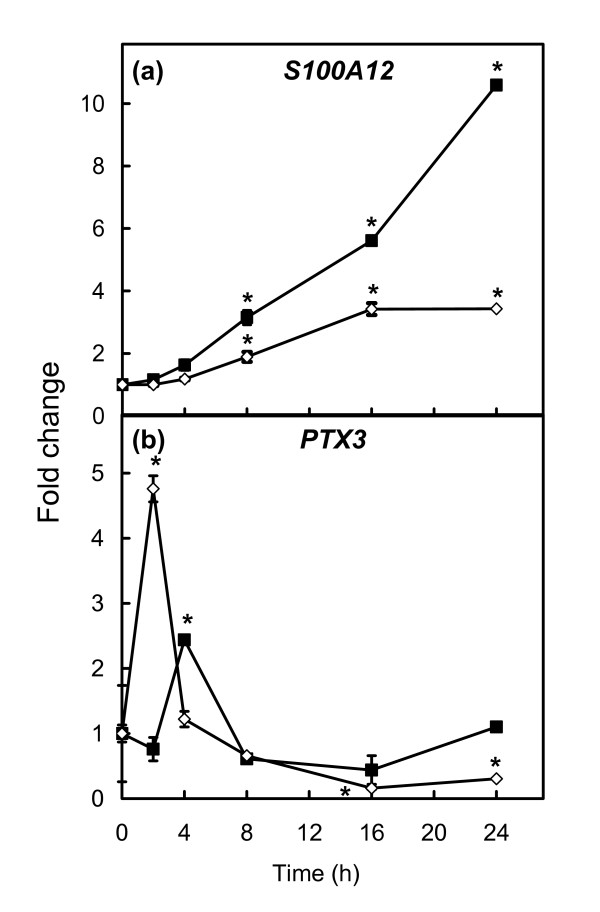
**Time-courses of *S100A12 *and *PTX3 *expression in bMEC stimulated with LPS and LTA**. bMEC were stimulated with either 50 μg/ml LPS (■) or 20 μg/ml LTA (◇) and the expression levels of *S100A12 *(a) and *PTX3 *(b) were monitored using qRT-PCR over a 24 h period. Data are expressed as mean fold change (*n *= 3) compared to expression in unstimulated cells. Significance was determined by ANOVA compared to unstimulated bMEC. P < 0.01 (*) was considered significant.

The expression levels of *S100A12 and PTX3 *transcripts in primary bovine mammary epithelial cells (bMEC) grown in cell culture were significantly increased in response to stimulation by 50 μg/ml of lipopolysaccharide (LPS) or 20 μg/ml of lipoteichoic acid (LTA) for a period of 24 h (Fig. 4). *S100A12 *was significantly (P < 0.01) up-regulated 4–24 h after LPS stimulation with a maximal fold change of 10.6 at 24 h. The effect of stimulation with LTA was less pronounced and resulted in significantly (P < 0.01) increased *S100A12 *mRNA expression at 8–24 h post-stimulation with the expression reaching a plateau at 16 h (Fig. 4(a)). *PTX3 *was up-regulated at 4 h (2.2 fold) of stimulation with LPS (P < 0.01) but then declined to basal levels. LTA stimulation resulted in enhanced expression of *PTX3 *at 2 h post-stimulation (4.5 fold) (P < 0.01) but the levels thereafter rapidly declined and at 16 h and 24 h there were significantly (P < 0.01) lower levels of expression compared to the starting sample (Fig. 4(b)). Thus, bMEC were likely to be the origin of at least some of the enhanced expression of *S100A12 *and *PTX3 *observed in mammary tissue challenged with *S. aureus*. Moreover, the time course responses for S100A12 and PTX3 were strikingly different.

### Expression and purification of rS100A12 and rPTX3

**Figure 5:**
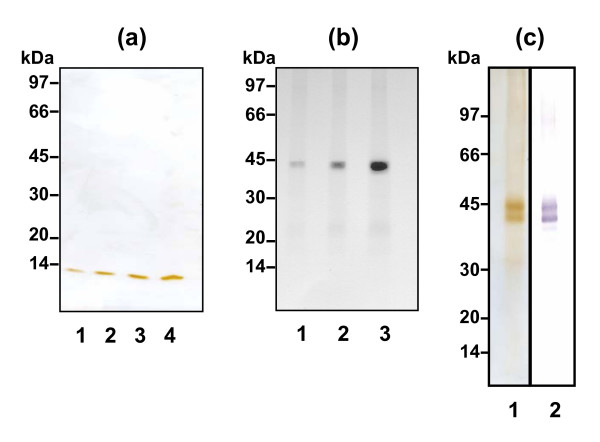
**Purified S100A12 and PTX3 recombinant proteins**. (a) SDS-PAGE analysis of rS100A12 purified using Ni-NTA affinity chromatography. All samples were run under reducing conditions. Lanes 1–4: 1, 2.5, 5 and 12.5 μg of rS100A12, respectively. (b) SDS-PAGE analysis of rPTX3 purified using Ni-NTA affinity chromatography. The samples were reduced and alkylated, and run under reducing conditions. Lanes 1–3: 1, 2 and 4 μg of rPTX3, respectively. (c) SDS-PAGE (lane 1) and immunoblot (lane 2) analyses of purified rtnPTX3 secreted by *Trichoplusia ni *cells. The samples were reduced and alkylated and run under reducing conditions. 10 μg of rtnPTX3 was used for the SDS-PAGE analysis and 5 μg for the immunoblot.

The coding sequences for bovine *S100A12 *and the mature form of bovine *PTX3 *were amplified by PCR, sequence verified and cloned into the pQE9 vector. The respective recombinant proteins, rS100A12 and rPTX3, were solubilised from inclusion bodies using 8 M urea and purified using Ni-NTA affinity chromatography. SDS-polyacrylamide gel analyses confirmed that the purified protein monomers were of the predicted sizes, i.e. ~11 kDa for rS100A12 and ~40 kDa for rPTX3 (Fig. 5). The latter protein was reduced and alkylated before analysis as this was required to ensure removal of rPTX3 oligomers. N-terminal sequencing further validated the identities of the two recombinant proteins. These recombinant proteins were used to raise corresponding polyclonal antibodies in rabbits, which were used to test whether S100A12 and PTX3 were present in milk samples from infected cows.

### Identification of S100A12 and PTX3 in milk from cows with intramammary S. aureus infection

**Figure 6:**
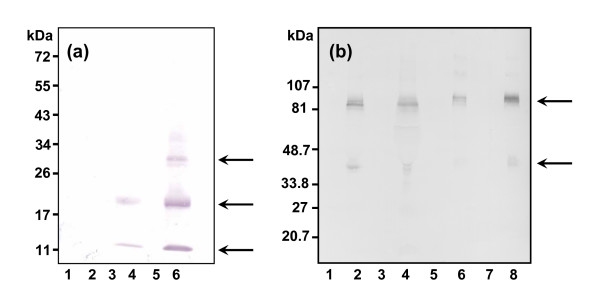
**Immunoblot analyses of S100A12 and PTX3 expression in milk whey from *S. aureus *infected cattle**. Representative immunoblot analyses of milk whey proteins before and after infection of mammary tissue with *S. aureus*. Each sample comprised 25 μl of milk whey and was run under reducing conditions. (a) Representative analysis of S100A12 expression in milk whey from cow 1419. Lane 1, control quarter pre-infection; lane 2, control quarter post-infection; lane 3, pre-infection control from the quarter receiving 1 × 10^5 ^*S. aureus*; lane 4, post-infection sample (1 × 10^5 ^bacteria); lane 5, pre-infection control from the quarter receiving 1 × 10^6 ^*S. aureus*; lane 6, post-infection sample (1 × 10^6 ^bacteria). Arrows denote the positions of S100A12 immunoreactive oligomers (monomer, dimer, trimer) detected using the immunoaffinity purified antibodies raised to rS100A12. (b) Analysis of PTX3 expression in milk whey. Lane 1, cow 1419 pre-infection sample from the quarter receiving 1 × 10^5 ^*S. aureus*; lane 2, post-infection sample (1 × 10^5 ^bacteria); lane 3, cow 1419 pre-infection sample from the quarter receiving 1 × 10^6 ^*S. aureus*; lane 4, cow 1419 post-infection sample (1 × 10^6 ^bacteria); lane 5, cow 1490 pre-infection sample from the quarter receiving 1 × 10^5 ^*S. aureus*; lane 6, cow 1490 post-infection sample (1 × 10^5 ^bacteria); lane 7, cow 1592 pre-infection control from the quarter receiving 1 × 10^5 ^*S. aureus*; lane 8, cow 1592 post-infection sample (1 × 10^5 ^bacteria). Arrows denote the positions of PTX3 immunoreactive bands (monomer and dimer) detected using immunoaffinity purified antibodies raised to rPTX3.

Figure 6 shows immunoblots of milk whey taken from control udder quarters or udder quarters infected with *S. aureus *at doses of 1 × 10^5 ^and 1 × 10^6 ^bacteria for 16 h. The antibodies used were immunoaffinity-purified antibodies raised to purified rS100A12 or rPTX3. In each case there was little or no detectable immunoreactivity in the milk whey samples from the uninfected controls or samples from udder quarters taken prior to the experimental infections. S100A12 and PTX3 immunoreactive bands were identified in all samples obtained from the infected quarters of the three tested cows. For cow 1419, three S100A12 immunoreactive bands were detected corresponding to monomer (Mr ~11 kDa), dimer (Mr ~22 kDa) and trimer (Mr ~33 kDa), despite the sample being reduced (Fig. 6(a)). The predicted size of bovine S100A12 is 10.7 kDa. The dimeric form was predominant. Human S100A12 exists as a highly stable dimer [42]. Similar results were also obtained for milk samples from cows 1490 and 1592. For PTX3, the predominant immunoreactive band was the dimeric form of PTX3 (Mr ~90 kDa), again despite the sample being run under reducing conditions. Monomeric PTX3 (Mr ~45 kDa) was also discernable (Fig. 6(b)). The predicted Mw of the mature PTX3 protein is 40.2 kDa. The bovine PTX3 predicted amino acid sequence contains one potential N-linked glycosylation site (Asn_221_) and indeed this type of glycosylation is present on the PTX3 protein derived from other species [43]. Close examination of the dimeric immunoreactive band reveals micro-heterogeneity that could reflect differential glycosylation or the formation of mixed intramolecular disulphide bonds. The latter could result from re-oxidation of cysteine residues during the electrophoretic separation process. For cow 1419 the immunoreactive bands were more intense at the bacterial dose of 1 × 10^6 ^compared to 1 × 10^5^. This result was consistent with the corresponding transcriptional changes (Fig. 1(c)) and measurements of the quantities of this protein in milk whey samples using an antigen capture ELISA (see below). The absence of S100A12 and PTX3 immunoreactivities in milk from both the control udder quarter and the pre-infection samples from cow 1419 indicates that the low level *S. aureus *infection detected in one (unused) udder quarter of this cow 24 h prior to the commencement of the experimental infection had not influenced the remaining quarters. Further, these data indicate that the experimental infection in one quarter did not influence the adjacent control udder quarter under these conditions.

### Quantification of S100A12 and PTX3 proteins in milk from cows subjected to intramammary infection with S. aureus

**Figure 7:**
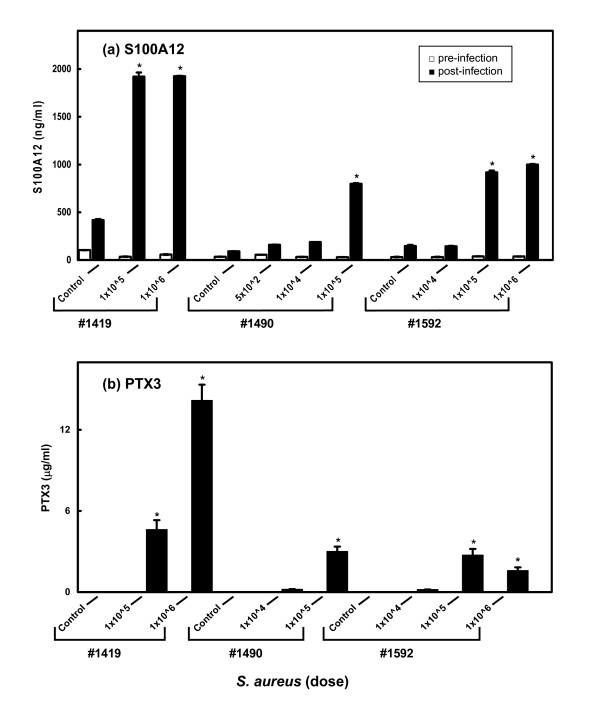
**S100A12 and PTX3 expression in milk from *S. aureus *infected cattle**. Antigen capture ELISAs were used to measure the concentrations of S100A12 (a) and PTX3 (b) in milk samples taken before and 16 h after the *S. aureus *intramammary challenge. Data from cows 1419, 1490 and 1592 were used in the analyses. Bars represent the means ± SEM for two independent samples. The asterisk denotes significant expression difference (1 way ANOVA, P < 0.01) compared with the corresponding post-infection control sample for each cow. No PTX3 expression could be detected in milk from the intra-animal control udders or in the pre-infection samples.

Antigen specific enzyme-linked immunosorbent assays (ELISAs) were developed to measure the quantities of S100A12 and PTX3 in milk samples taken from udder quarters after *S. aureus *intramammary challenges of the three cows (Fig. 7). Significantly increased S100A12 protein expression (P < 0.01) was detected in the milk whey from the infected udders in all three cows relative to their respective intramammary control quarters (at 16 h post-infection). There was a low level of S100A12 expression in milk samples from the control non-infected udders. Moreover, there was a relatively small but significantly (P < 0.01) higher level of S100A12 in milk from the control udder quarters 16 h post-infection compared with the corresponding pre-infection samples. Thus, using this antigen capture ELISA there was evidence for a small intermammary quarter effect on S100A12 levels in milk.

The responses to infection in the different cows were varied. Cow 1419 had a concentration of 420 ng/ml of S100A12 in milk from the control udder at 16 h post-infection while the S100A12 levels in the udders that had been challenged with 1 × 10^5 ^and 1 × 10^6 ^bacteria were almost five times greater (1920 ng/ml in the milk from both udder quarters) (P < 0.01). Cow 1490 had lower levels of S100A12 in the control udder (90 ng/ml) and the levels of S100A12 increased in a dose-dependent manner (P < 0.01). The S100A12 protein expression was relatively high in milk from udder quarters infected with bacterial doses of 1 × 10^5 ^and 1 × 10^6 ^in cow 1592 (918 ng/ml and 1000 ng/ml, respectively) (P < 0.01) while the quantities of S100A12 detected in milk from the udder quarter challenged with the lowest dose of 1 × 10^4 ^bacteria (140 ng/ml) was not significantly different from the control udder (150 ng/ml).

Increased concentrations of PTX3 were detected in milk whey from the infected udders in all three cows compared to their intramammary quarters (Fig. 7(b)). PTX3 could not be detected in milk from the pre-infection udders or the post-infection control udders. Cow 1419 showed the highest levels of PTX3 in milk from the udder quarters that had been challenged with 1 × 10^5 ^and 1 × 10^6 ^bacteria (4.6 μg/ml and 14.2 μg/ml, respectively). Cow 1490 had less marked, but still significantly (P < 0.01) increased levels of PTX3 in response to the bacterial dose of 1 × 10^5 ^(i.e. 3 μg/ml). The quantities of PTX3 detected in milk from the udder quarters of cow 1592 challenged with the two higher bacterial doses, 1 × 10^5 ^and 1 × 10^6 ^of *S. aureus*, were significantly (P < 0.01) increased (i.e. 2.7 μg/ml and 1.6 μg/ml respectively).

### Correlation between the Somatic Cell Count (SCC) and S100A12 protein expression in milk from dairy cows

**Figure 8:**
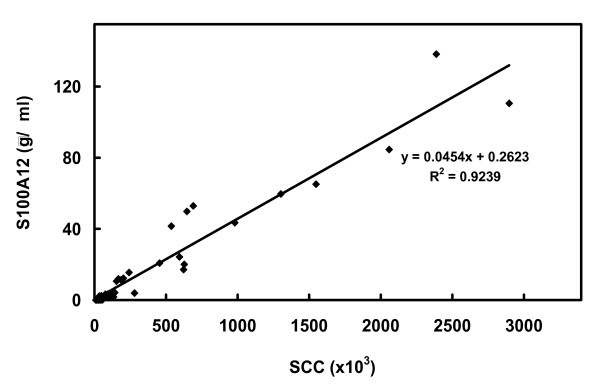
**Correlation between the Somatic Cell Count (SCC) and S100A12 concentration in milk from dairy cattle**. The plot shows the correlation between S100A12 concentration and SCC in milk from 38 dairy cows. The line of best fit drawn through the data has a correlation coefficient (R^2^) of 0.92. The SCC was determined by flow cytometry of milk samples and the S100A12 levels in milk whey were determined using the S100A12-specific antigen capture ELISA with rS100A12 as the standard.

The S100A12 protein levels in milk were measured using the S100A12-specific antigen capture ELISA and correlated with the milk somatic cell count (SCC) from a selection of 38 dairy cows with SCC ranging from 1 × 10^4 ^– 3 × 10^6 ^cells/ml (Fig. 8). There was a linear correlation between S100A12 quantity in milk and milk SCC (R^2 ^= 0.92). The PTX3-specific antigen capture ELISA was not used to examine the correlation between PTX3 quantity in milk and SCC due to the relatively low levels of PTX3 present in all milk samples.

### Recombinant S100A12 has anti-microbial activity against E. coli

**Figure 9:**
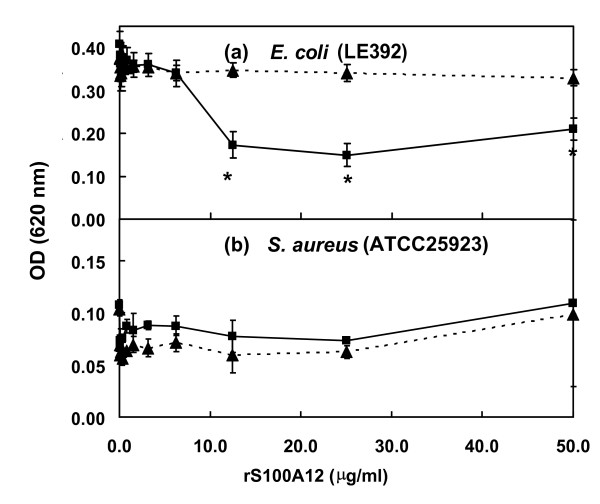
**Antimicrobial activity of rS100A12**. Antimicrobial activities of rS100A12 were measured using the Broth Microdilution Susceptibility Testing Method. (a) *E. coli *and (b) *S. aureus*. (■), rS100A12; (▲) BSA at the same concentrations. Bars represent the means ± SEM for 2 replicates. The data were examined for significance using ANOVA and the asterisk denotes significant differences (P < 0.05) from the control.

A previous publication suggested that human S100A12 may have anti-microbial activity [44]. The capacity of recombinant bovine S100A12 (rS100A12) to inhibit *E. coli *growth was examined using a microtiter dilution anti-microbial susceptibility testing assay [45]. Inhibition of growth of *E. coli *(strain LE392) was observed in the rS100A12 concentration range of 12.5–50 μg/ml (P < 0.05) (Fig. 9(a)). The greatest inhibitory activity was observed using 25 μg/ml of rS100A12, which resulted in a 2.3 fold reduction in optical density of the *E. coli *culture. Under the same conditions rS100A12 did not significantly (P > 0.05) affect the growth of S. aureus (ATCC 25923) at any concentration (Fig. 9(b)).

### Expression of recombinant bovine PTX3 in Trichoplusia ni insect cells

Recombinant bovine PTX3 (rtnPTX3) was also produced in insect cells to obtain a correctly folded and biologically active protein. The culture supernatant from the *Trichoplusia ni *(High Five™) insect cells transfected with the pIEX5-PTX3 construct was analysed by SDS-PAGE and immunobloting under reducing conditions. Immunoreactive proteins with an apparent molecular size of 45 kDa (monomer) and 90 kDa (dimer) were present but not observed in the culture supernatant from the plasmid-only control (results not shown). rtnPTX3 produced by these cells was purified from the culture supernatant using anion exchange chromatography. Figure 5(c) shows silver-stained SDS-PAGE and immunoblot analyses of purified rtnPTX3 after reduction and alkylation. The purified protein was represented by two equal staining bands of Mr 43 kDa and 45 kDa compared with a predicted size for the mature protein of 40.2 kDa. The PTX3 sequence has a potential *N*-linked glycosylation site at amino acid position 221 (N_221_) and hence the larger band may reflect a more glycosylated form of the protein. N-terminal sequencing of rtnPTX3, from both bands, showed identity with the predicted amino acid sequence of the mature bovine PTX3 polypeptide (GenPept accession AAI20176). The mature PTX3 protein sequence contains nine cysteine residues, which may mediate formation of multimers [43]. Indeed, rtnPTX3 was present in solution as a large oligomer (decamer) as defined by gel permeation chromatography (result not shown).

### Purified rtnPTX3 binds the C1q component of the human complement system

**Figure 10:**
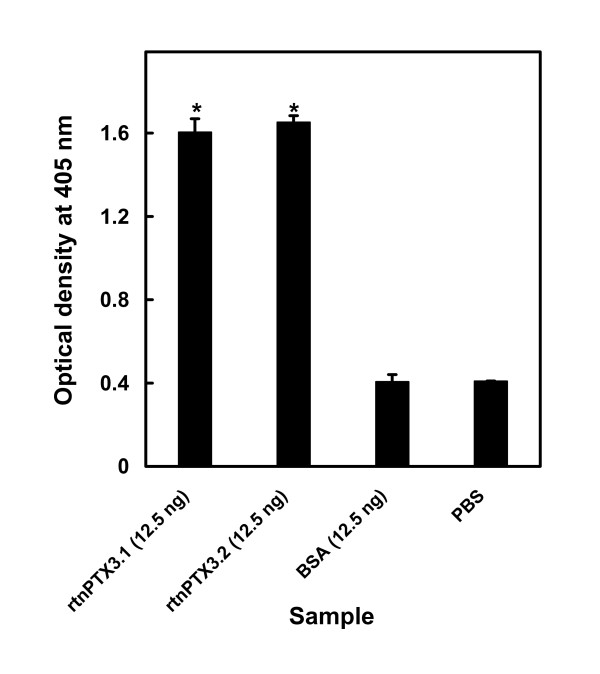
**Binding of rtnPTX3 to immobilised human C1q**. The binding of rtnPTX3 to immobilised human C1q was measured using an ELISA-based binding assay. The binding activities for two different preparations of rtnPTX3 (each 12.5 ng/well) are shown. The controls consisted of PBS and BSA (12.5 ng/well). Significance was measured by ANOVA relative to the PBS control (*, P < 0.05).

The binding of human PTX3 expressed in Chinese Hamster Ovary (CHO) cells to human C1q has been previously demonstrated [43,46]. To characterise the binding of rtnPTX3 to C1q, dilutions of two different preparations of purified rtnPTX3 were added to 500 ng of immobilised human C1q and after washing bound rtnPTX3 was measured. Figure 10 demonstrates that rtnPTX3 bound to human C1q compared to either the BSA or PBS control (Fig. 10).

### rtnPTX3 binds E. coli

**Figure 11:**
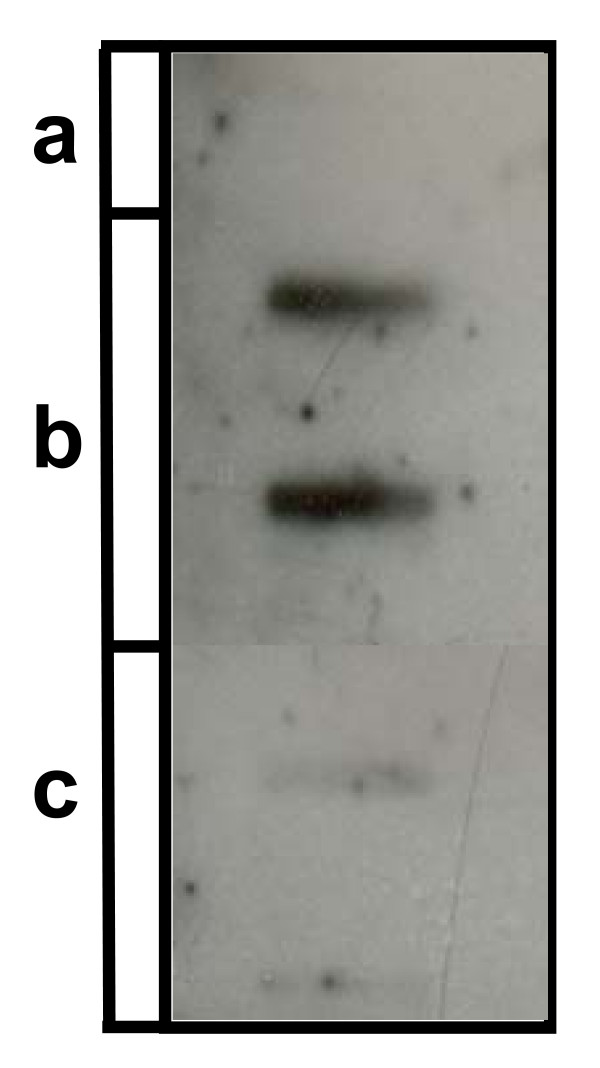
**Direct binding of biotinylated *E. coli *to rtnPTX3**. The binding of biotinylated *E. coli *(ATCC 35218) to rtnPTX3 immobilised onto a nitro-cellulose membrane was measured. (a) PBS; (b) rPTX3 in duplicate (50 μg); and (c) BSA, in duplicate (50 μg).

rtnPTX3 was tested for ability to inhibit the growth of *E. coli *(strain LE392) or S. aureus (ATCC 25923) using the assay described for rS100A12. No direct activity could be demonstrated. However, it has been reported that human PTX3 binds selected pathogens including *Pseudomonas aeruginosa*, *Salmonella typhimurium *and *Aspergillus fumigatus *[47]. To investigate the possibility that bovine rtnPTX3 binds *E. coli*, the protein was bound to nitrocellulose in a slot blot and then probed with biotinylated *E. coli*. The slot blot showed that biotinylated *E. coli *in PBS bound to rtnPTX3 but not the same quantity of BSA (Fig. 11). Biotinylated *S. aureus *(1 × 10^9 ^bacteria) in PBS did not bind to the immobilised rtnPTX3 (result not shown).

## Discussion

The objective of this study was to characterise the host transcriptional response to intra-mammary infection with *S. aureus*, a Gram-positive bacterial pathogen. We initially investigated the mRNA expression levels of two major milk protein genes (*CSN1S1 *and *CSN2*) in the udder tissue to confirm that transcripts encoding proteins involved in the major physiological function of the udder, milk protein production, were not affected by the experimental infection. These qRT-PCR measurements demonstrated that the limited experimental infection, in terms of infection time and dose, had not caused significant loss of function in the mammary tissue. Thus, the experimental model measured changes in immune gene responses that are likely to be appropriate for the initial stages of infection.

Transcript profiling was employed to analyse gene expression in mammary tissue challenged by *S. aureus *infection. Initially, mRNA levels of a number of genes (*CD14*, *IL-1β*, *IL-6*, *IL-8 *and *TNFα*), principally encoding proinflammatory cytokines, were measured. These genes showed significant dose-dependent up-regulation in all three cows subjected to intramammary infusion with *S. aureus *for a 16 h period. The dose dependent transcriptional responses probably directly reflect the dose of bacteria in the tissue and also, due to the lag phase in bacterial growth, different durations of inflammation in the tissue. This experiment also demonstrated that *S. aureus *intramammary infusion had generated an immune response that was self-contained within each udder quarter i.e. the experimental infection in one udder quarter did not affect gene expression in the adjacent control udder quarter. Although *TNFα *mRNA, measured by qRT-PCR, was up-regulated by the intramammary infusion of *S. aureus*, it has been reported that the protein was not detectable in milk from udders infected with *S. aureus *[48,49]. One possible explanation is that this apparent disparity reflects the differing sensitivities and dynamic ranges of the measurement technologies. Another possibility is that the observed changes in mRNA expression for TNFα do not accurately translate into corresponding secreted protein changes. This could occur when mRNA translation or protein secretion were rate limiting in defining the quantity of TNFα in milk.

The reasons for the more accentuated responses in cow 1419 are not clear but may reflect differing genetics underlying individual immune responsiveness or a different history of disease exposure. It is also possible that cow 1419 had been primed for a stronger innate immune response as a result of a low level of asymptomatic pre-existing infection in one quarter prior to the experimental challenge, despite this quarter not being used in the experiment. However, the control quarter from this cow did not display increased expression of *TNFα *or *IL-8 *relative to the controls from the other two cows, indicating that expression of genes in the control quarter from cow 1419 was not systemically affected by a pre-existing mild infection in the unused adjacent quarter. Another possibility is that the infection was not uniform in the udder quarter and sampling had occurred in regions from this animal that had a greater extent of infection.

Previous studies have clearly demonstrated that mammary epithelial cells have a robust innate immune capability and thus the observed responses in infected mammary tissue probably reflect transcriptional changes in these cells as well as cellularity changes caused by infiltrating immune cells [12]. It might be expected that the predominant responses would be from the mammary epithelial cells at low infection rates and infiltrating immune cells at the higher doses as they respond to proinflammatory signals generated by the former cells. The relative dissection of these contributions awaits further investigation possibly involving immunolocalizations or in situ mRNA localizations.

Microarray transcript profiling identified two major clusters of differentially expressed genes: genes that were up-regulated and down-regulated after infusion of *S. aureus *into the mammary gland. The majority of the up-regulated genes encoded proteins involved in intercellular signalling and a large number of them were cytokines and chemokines (e.g. CXCL1, CXCL2, CXCL10, IL-1β, IL-6 and IL-8). These proteins are intimately associated with inflammatory processes and are probably essential for the recruitment and activation of neutrophils into the infected mammary tissue [12,27,50]. The overflow of these cells into milk is a widely used marker of mastitis in dairy cows. The down-regulated genes encoded components of the cytoskeleton or extracellular matrix, mediators of apoptosis, cell surface receptors and intercellular signalling proteins. The former group suggests that tissue remodelling of the mammary tissue in response to infection was beginning to occur despite the maintenance of normal milk protein gene expression.

The up-regulated cluster also contained genes encoding proteins with less defined functions. Two such genes, *S100A12 *and *PTX3*, were investigated in greater detail as they have been previously implicated in inflammatory responses [51,52] although their specific roles particularly in relation to bovine mammary tissue have not been defined. *S100A12 *and *PTX3 *mRNAs were markedly up-regulated in bMEC stimulated with LPS and LTA indicating that they may be involved in the initial response of mammary tissue to bacterial infection. However, the time courses of their responses were very different with PTX3 showing earlier up-regulation but unlike S100A12 it did not show a sustained response. Thus, the time course responses of these two genes to the infection were dissimilar.

The S100A12 protein (also known as calgranulin C) triggers a signal transduction cascade that results in activation of the transcriptional co-regulator NF-κB and consequent expression of pro-inflammatory cytokines [32,53]. Increased expression of S100A12 in bMEC could amplify the pro-inflammatory response in infected mammary tissue and thereby promote the infiltration of neutrophils into the tissue. S100A12 is a small (92 amino acids) calcium-binding protein. Interestingly, its mRNA sequence does not encode a signal sequence even though the protein is present externally to stimulated cells and there is a cell surface receptor, Receptor for Advanced Glycation End products (RAGE), for S100A12 on the surface of many cells [32]. One possibility is that S100A12 is released from cells by an undefined secretory mechanism. Another possibility is that it is released from cells undergoing inflammation-induced cell death. The interaction between S100A12 and RAGE is thought to amplify and propagate the inflammatory response in endothelial cells through increased NF-κB activation and expression of pro-inflammatory cytokines. Blockage of this interaction dampens the response by inhibiting the nuclear translocation of NF-κB [42]. RAGE is a multi-ligand member of the immunoglobulin superfamily of cell surface molecules that binds to Advanced Glycation End products (AGEs), proinflammatory cytokine-like mediators of the S100/calgranulin family, as well as amphoterin and amyloid-β-protein [54]. The presence and distribution of RAGE in mammary tissue is unknown.

S100A12 was present in milk samples from *S. aureus*-infected udder quarters and the level of the protein was dependent on the severity of infection. Relatively low quantities of S100A12 were also found in the milk samples taken prior to the *S. aureus *challenge. Interestingly, using an antigen capture ELISA it was demonstrated that S100A12 levels in milk samples from the control udder quarters at 16 h post-infection showed a small but significant increase compared to the pre-infection control samples. Thus, there was a small intermammary quarter effect of the infection but this was minor compared to the responses in the infected quarters. In a dairy herd there was significant correlation between the level of milk S100A12 and SCC, a widely accepted indicator of mastitis. Thus, S100A12 protein levels in milk could be used as an indicator of mastitis in dairy cattle.

The current study has also demonstrated that rS100A12 inhibited the growth of *E. coli *but not *S. aureus *in a dose-dependent manner (Fig. 9). This novel finding suggests that S100A12 may have a direct role in protecting mammary tissue from infection by coliform bacteria and may play a role in protecting newborn suckling calves from bacterial infections. The detailed nature of this inhibitory activity is yet to be defined. The result is somewhat paradoxical in view of the up-regulation of the expression of this gene in bMEC stimulated with LTA, a Gram positive bacterial cell wall component as well as LPS, a Gram negative bacterial cell wall component.

The long pentraxin, PTX3, is a secreted multimeric glycoprotein that is thought to activate the immune system by directly binding selected micro-organisms and C1q, the first component of the classical pathway of complement activation [43,47]. Indeed, PTX3 is a secreted pattern recognition receptor. The expression of bovine PTX3 mRNA was strongly and rapidly up-regulated in bMEC challenged with LPS and LTA and increased in mammary tissue from udders infected with *S. aureus*. Thus, PTX3 has the characteristics of an innate immune protein. Although no direct anti-bacterial activity could be demonstrated, rtnPTX3 protein was able to directly bind *E. coli*. This result is consistent with a pattern recognition function of PTX3 that facilitates the immobilisation and display of micro-organisms. The decameric structure of PTX3, particularly its disulphide bonded structure, is thought to underpin this activity [31,43]. The bovine recombinant protein also underwent oligomerisation (results not shown). In addition, the current study demonstrated that recombinant bovine PTX3 bound human C1q, thereby physically linking the complement system with PTX3-bound bacteria, presumably promoting opsomization of the bacteria. At peak lactation only the alternative complement pathway is active in milk due the absence of C1q. Therefore the actions of PTX3 probably operate only after inflammatory exudation of plasma components into milk [55]. The relatively rapid transcriptional response of bMEC to LTA is consistent with the view that PTX3 is a component of the immediate early response, where it acts to initially bind and coat bacteria, and subsequently activates the classical pathway of the complement system.

## Conclusion

Bovine S100A12 probably amplifies the inflammatory response via recruitment of neutrophils while bovine PTX3 activates the immune system by binding to C1q and selected pathogens. Acting in concert, these proteins are likely to be important components of a multi-faceted response of mammary tissue to infection. These proteins may lead to novel therapeutic strategies for the treatment and prevention of mastitis. Since S100A12 and PTX3 display microbial growth inhibition and binding properties, respectively, they could provide a new class of natural anti-microbial agents that could assist defense of the mammary gland against chronic and subclinical infections. The presence of these innate immune signalling proteins, amongst other cytokines, in milk may also be instructive for the maturation of the neonatal immune system and gut epithelium as well as influencing microbial colonisation of the neonatal gut.

## Methods

### Cows

Three multiparous Holstein Friesian cows at the peak of their third or fourth lactation cycles and approximately 21 days post parturition were used. These cows had the following characteristics: Australian Breed Value (ABV) in the top 25% ; lactation 5,500–9000 L/year; no history of clinical mastitis; SCCs < 200,000 cells/ml; and Johne's disease free. Each cow represented a different sire line and was fed and housed according to standard operating procedures at the Camden Campus, University of Sydney. None of the cows had any clinical signs of mastitis in any quarter prior to the experiment (i.e. no swelling, no overt signs of pain after prodding the udder, no redness, no clots in their milk, SCCs < 200,000 cells/ml, no bacterial growth in milk samples). Animal experiments were performed in accordance with Australian Animal Ethics Guidelines and with approval from the Faculty of Veterinary Science Animal Ethics Committee, University of Sydney (N00/10-2003/2/3829: Collection of Mammary Biopsies for Dairy Cattle: the Induction of Mastitis).

### S. aureus strain

An isolate of the virulent *S. aureus *strain, JG-80 [56], derived from a clinical case of ovine mastitis was grown overnight at 37°C in nutrient broth (BO0759Z; Oxoid). Bacteria were pelleted (3,600 × *g*, 4°C, 15 min) and washed in 20 ml of pyrogen-free PBS (×3). The bacterial concentration was estimated using the conversion ratio: OD_600 _of 0.46 equals 1 × 10^9 ^bacteria/ml [57]. Bacterial cultures were adjusted to the desired concentration by dilution in 5 ml of pyrogen-free PBS.

### S. aureus intramammary challenge and mammary tissue collection

The udders of the cows were determined to be free of bacteria by assaying a 10 ml sample of milk from each quarter for bacterial growth, 24 h and 48 h prior to infusion with *S. aureus*. Despite the complete absence of any clinical symptoms and a SCC < 50,000, one quarter of cow 1419 was found to have a low level *S. aureus *infection in the milk sample that was collected 24 h but not 48 h prior to the commencement of the experiment. As a consequence, this quarter was excluded from the experiment. Immediately following morning milking the udders were washed and disinfected. One quarter of the udder on each cow was infused with 5 ml of pyrogen-free PBS (intra-animal control), while the other three quarters of each cow (except cow 1419) received a graded dose of *S. aureus *(5 × 10^2 ^– 1 × 10^6 ^bacteria) (Table 1). Infusions were delivered using a 4 cm infusion cannula inserted into the teat canal and then massaged further along the teat canal and into the udder tissue. The doses of bacteria were designed to produce low level subclinical mastitis (i.e. SCC > 200,000 cells/ml) within 16 h after which a second sample of milk (10 ml) was collected for diagnostic analysis before the cows were euthanized. A 10–20 g sample of mammary tissue was then surgically removed from each udder quarter in less than 10 min, snap frozen in three segments in liquid nitrogen and stored at -80°C. The secretory tissue samples were taken from carefully selected regions of each udder quarter avoiding large blood vessels and the cisternal region.

### Diagnostic analyses of milk samples

Milk was collected according to a standard protocol [58] before and after pathogen infusion and sent for analysis to the Veterinary Pathology Diagnostic Services (University of Sydney). Standard procedures were used for assessment of bacterial content. The milk samples were assayed for bacterial growth and colony characteristics, and cultured bacteria were characterised using the Gram stain [59]. The bacterial culture was identified and tested for haemolytic, catalase and coagulase activities [60]. *S. aureus *was identified in cultures obtained from milk samples using the following criteria: growth and colony characteristics on Sheep Blood agar from Oxoid (Oxoid Australia Pty Ltd., Victoria); Gram positive stain; haemolytic on blood agar medium; catalase positive and coagulase positive. SCC was determined using laser based flow cytometry of the milk samples.

### Preparation of RNA

Frozen mammary tissue (~3 g) was placed in liquid nitrogen, pulverised with a hammer, followed by addition of 25 ml of Trizol reagent (Invitrogen). The tissue was homogenised using an Ultraturrax probe (IKA, 3 cycles of 2 s) and RNA extracted according to the manufacturer's protocol (Invitrogen). RNA was further purified using an RNeasy Midi Kit (Qiagen) and each sample was treated twice with DNase I to eliminate contaminating genomic DNA. The purified RNA was quantified by spectrophotometric measurements and its purity and integrity verified by the OD_260_/OD_280 _ratio ( > 1.8) and by visualisation on a denaturing gel. Controls without reverse transcriptase were also produced for qRT-PCR analyses to validate the absence of genomic DNA.

### Primer design and quantitative real time RT-PCR (qRT-PCR)

All gene symbols are shown in italics when used in the context of the gene or mRNA but when used in the context of their encoded proteins they are denoted by normal text. Quantitative Real-Time Reverse Transcription PCR (qRT-PCR) using the Sybr Green-based fluorescent detection system and the ABI Prism 7900 Sequence Detection System (PE Applied Biosystems, Forster City, CA), was used to measure mRNA abundance. Analysis of the data was performed using the Relative Expression Software Tool (REST), which generates a Mean Normalised Expression (MNE) value of a target transcript relative to a reference gene [61,62]. Primers were designed with the aid of MacVector (ver 7.1) (Accelrys) software and publicly available ovine and bovine sequences (Table 5). A constant amount of cDNA (10 ng) was used for qRT-PCR measurements. Four technical replicates were performed for each gene investigated. In addition, three independent cDNA samples were assessed for each tissue sample from each cow. This process allowed quantification of the target gene relative to a constant reference gene in each sample using threshold cycle (Ct) data. Three potential reference genes, *18S Ribosomal RNA *(Genbank accession DQ222453), *Glyceraldehyde 3-Phosphate Dehydrogenase *(Genbank accession XM_868165) and *Ribosomal Protein, large, P0 *(*RPLP0*) (Genbank accession BT021080) were independently measured in all samples at constant cDNA input to determine which was the most suitable reference gene. This analysis revealed that the expression pattern of *RPLP0 *showed least variation and therefore it was used as the reference gene.

**Table 5:** Oligonucleotide primer sequences used for qRT-PCR and recombinant protein expression

**Gene**	**Accession number**	**Forward (5'→3')**	**Reverse (5'→3')**	**Size (bp)**	**Ref**.
*RPLP0*	BT021080	CAACCCTGAAGTGCTTGACAT	AGGCAGATGGATCAGCCA	220	[12]
*IL-1β*	M35589	AAATGAACCGAGAAGTGGTGTT	TTCCATATTCCTCTTGGGGTAGA	185	[12]
*IL-6*	X57317	CTGGGTTCAATCAGGCGAT	CAGCAGGTCAGTGTTTGTGG	206	[12]
*IL-8*	AF232704	CTGTGTGAAGCTGCAGTTCT	TAAGCAGACCTCGTTTCCAT	180	[12]
*TNFα*	NM_173966	CTGGTTCAGACACTCAGGTCCT	GAGGTAAAGCCCGTCAGCA	183	[12]
*CD14*	D84509	GGTGCTACCCGATGTGTCTG	AAGGGATTTCCGTCCAGAGT	191	[12]
*CSN1S1*	M38641	CCTGTCTTGTGGCTGTTGCTCTTG	CATCTTCCTTTTGAATGTGCTTCTGCT	282	pc*
*CSN2*	M16645	TCTGCCTCTGCTCCAGTCTT	AGGAGGGGGCATTCACTTT	251	pc*
*S100A12*	NM_174651	CATTTCGACACCCTCAACAA	CTGTTTTCAGCACCCTGGAC	184	
rS100A12	NM_174651	CCGGATCCACTAAGCTGGAAGATCACCTGGAGG	CCAAGCTTTACTCTTTGTGGATATCTATGTGGGCTG	-	
*PTX3*	NM_001076259	TATGCCATGGTGCTTTCAGA	CCAATGAACAATGGACAACAA	182	
rPTX3	NM_001076259	ATGGATCCGAGAACTCAGATGATTATGAGCTCATGTA	AGAAGCTTTTAATAAACATACTGTGCTCCTCGTG	-	
rtnPTX3	NM_001076259	GTGGATCCGGAGAACTCAGATGATTATGAGCTCATGTA	AGAAGCTTTTAATAAACATACTGTGCTCCTCGTG	-	

* Personal communication (Karen Matthews)

Each qRT-PCR (5 μl) contained: 2.5 μl of 2× Sybr Green Master Mix (Applied Biosystems); 0.25 μl of each primer giving a final concentration of 450 nM each; 1.0 μl water; and 1.0 μl of a 1/10 dilution of the stock cDNA template. The reaction was started by heating (95°C, 10 min) to activate the AmpliTaq Gold DNA polymerase. The cycling conditions consisted of 40 cycles of 95°C for 15 s and 60°C for 1 min. All melt curves showed a single peak consistent with the presence of a single specific amplicon. Each qRT-PCR assay was validated by amplicon size and sequence. Amplification efficiency was independently measured for each amplicon [61]. Udder quarter differences in gene expression for each cow were analysed using One-way ANOVA and P < 0.01 was considered statistically significant.

### cDNA microarray transcript profiling

The microarray experiments were performed using the Bovine Innate Immune Microarray platform [28]. The cDNA microarray comprises 1480 defined immune related genes in duplicate and 5327 anonymous cDNAs (in duplicate) from subtracted and normalised cDNA libraries derived from bovine peripheral blood lymphocytes (PBL), bovine mammary epithelial cells (MAC-T and bMEC) and bovine macrophages (BoMac), which underwent a variety of immune challenges. In addition, the microarray contains 384 control elements in quadruplicate and 49 orthologous ovine/bovine elements in quadruplicate. In some cases a single gene is represented by multiple elements. cDNA synthesis, labelling, microarray hybridisation and washing, and data acquisition were performed as previously described [28]. Data from each cow were separately analysed relative to their intramammary control as the initial qRT-PCR studies indicated that there was considerable variation in the magnitudes of individual cow responses for each tested gene. In the microarray analysis, gene expression in each quarter within a cow was directly compared to the gene expression in other quarters using an 'all pairs' design with reciprocal dye swaps i.e. in general, 12 microarrays were used per cow and 6 measurements were made of each sample. In this manner, each cow was initially independently analysed (Fig. 2). Data from these microarray experiments has been submitted to the Gene Expression Omnibus [63] (platform ID GPL6082, Series ID GSE9685).

### Microarray statistical analysis

Statistical analysis of the microarray data was performed as previously described [28]. The mixed model used for the analysis of intensity expression signals accounted for 95.5% of the total variation. The main effect of probe and the interactions between probe by array, probe by dye and probe by dose accounted for 76.8%, 15.3%, 1.6% and 1.8%, respectively. These proportions are consistent with previous studies applying mixed models to the analysis of expression data and anticipate that, for an optimal control of false discovery rate, approximately 1.8% of probes are expected to be found differentially expressed in any given contrast. The overall effects of *S. aureus *infection on mammary gene expression were visualised using GeneSpring 5.1 (Silicon Genetics, Redwood City, CA). The mean signal intensity for each significantly differentially expressed element, calculated using the statistical model described above, was loaded into the software. Differentially expressed elements, but limited to those with greater than 2 fold change in at least one sample and expression in all samples, were identified by analysis of samples from individual cows. The mean signal intensity for each treatment dose was calculated and these values were normalised to the mean signal intensity for the element in the intra-animal control sample. Thus, each differentially expressed element had a normalised mean signal intensity value for each *S. aureus *dose i.e. 500 (*n *= 1), 10,000 (*n *= 2), 100,000 (*n *= 3) or 1,000,000 (*n *= 2) bacteria, compared to the respective control samples. The reasons for the different number of biological samples with the different doses related to a different infection regime for one cow and the exclusion of one quarter in another cow from the experiment. The resulting profiles were subsequently clustered (K-means clustering) [30]. However, manual examination of these clusters in conjunction with the inherent redundancy on the microarray revealed that they required considerable consolidation. Consequently, the clusters were manually condensed into two primary groups i.e. a cluster of elements showing *S. aureus*-induced increased expression and a cluster of elements showing *S. aureus*-induced decreased expression.

### Sequencing of cDNA clones

The Bovine Innate Immune Microarray comprised a significant number of elements derived from anonymous cDNA clones. The anonymous elements with expression profiles that were significantly altered by *S. aureus *infection were sequenced using standard protocols (Applied Biosystems, USA). The clipped EST sequences were submitted to the Genbank nucleotide sequence database with accession numbers EW739703–EW741108[64]. Annotations were added to the EST sequences by comparing them to the Genbank non-redundant and Human RefSeq nucleotide and amino acid databases using BLASTN and BLASTX [65,66]. Gene names were assigned to ESTs if the Expect score was less than 1 × 10^-10^. The predicted biological functions of these genes were then determined through extensive PubMed literature searches for ortholog functions [64].

### Challenge of bMEC with LPS and LTA

The culture and characterisation of the primary bovine mammary epithelial cells (bMEC) have been described elsewhere [12]. bMEC were grown in M199, Hams F12, 10 mM Hepes, 20 mM NaHCO_3_, 2 mM NaC_2_H_3_O_2_, 20% horse serum, 5% FCS, 100 U/ml penicillin, 100 μg/ml streptomycin, 250 ng/ml amphotericin B, 100 ng/ml kanamycin (all reagents from Invitrogen Corporation Carlsbad, California), 5 μg/ml insulin, 1 μg/ml cortisol (Sigma Chemical Co., St Louis), 10 ng/ml EGF (MP Biomedicals, Irvine, CA), pH 7.4. bMEC were seeded at 4 × 10^4 ^cells per well in 6 well plates (Sarstedt) and grown until 80% confluence at 37°C, then challenged with LPS (50 μg/ml) or LTA (20 μg/ml) for 0, 2, 4, 8, 16 and 24 h. The concentrations of LTA and LPS used with the bMEC were determined from titration analyses [12]. LPS was purchased as a lyophilised powder purified from *E. coli *strain 055:B5 by phenol extraction (Sigma Chemical Co.) [67]. LTA isolated from *Streptococcus pyogenes *was obtained from Sigma Chemical Co [68].

### Cloning, expression and purification of rS100A12 and rPTX3

The nucleotide sequence encoding bovine PTX3, without the region encoding the signal sequence, was cloned into the bacterial expression vector pQE9 (Qiagen) and expressed and purified according to the manufacturer's instructions (primer sequences are listed in Table 5). rPTX3 was identified by size, N-terminal sequence and its reactivity with the Ni-NTA affinity resin and Ni-NTA HRP conjugate. The protein was refolded [69] and dialysed into 50 mM Tris-HCl pH 8, 500 mM NaCl and 20 % glycerol.

The nucleotide sequence encoding bovine S100A12 was cloned into the bacterial expression vector pQE9 (Qiagen) (Table 5) and expressed as per the manufacturer's instructions. The recombinant protein (rS100A12) was expressed in the cytoplasm and purified using Ni-NTA affinity chromatography (Qiagen). All buffers contained Complete EDTA-free protease inhibitors (Roche Applied Science). Protein quantities were determined using the BCA Protein Assay kit (Pierce) with BSA as the standard. Endotoxin levels in the recombinant proteins were less than 0.05 EU/ml using the Cambrex Limulus Amebocyte Lysate (LAL) pyro Plus Gel Cot assay (lonza, Basel, Switzerland)

### N-terminal amino acid sequencing of rS100A12 and rPTX3

Purified rPTX3 and rS100A12 were blotted onto polyvinylidine fluoride (PVDF) membrane (Problot, Applied Biosystems) and their N-terminal amino acid sequences were determined using a Procise Protein Sequencer (Applied Biosystems). The sequences confirmed the expected N-terminal sequences (underlined) i.e. MRGSHHHHHHGS **ENSDDY**(rPTX3); MRGSHHHHHHGS **TKLEDHLE**(rS100A12).

### Production of polyclonal antibodies and development of S100A12 and PTX3-specific enzyme-linked immunosorbent assays (ELISAs)

Four aliquots, each of 50 μg of purified rPTX3 (in 6 M urea), and four aliquots, each of 250 μg of purified S100A12 (in PBS), were used for the production of rabbit polyclonal antibodies (Institute of Medical and Veterinary Science Polyclonal Antibody Production, Gilles Plains, South Australia). Each rabbit was injected with one aliquot of antigen (with adjuvant) 4 times at 3 week intervals. Serum samples were collected from the rabbits 2 weeks after the last injection. Antibodies to rPTX3 and rS100A12 were immunoaffinity purified [69]. Antigen-specific capture ELISAs were used to measure the concentrations of endogenous S100A12 and PTX3 in milk whey samples. The antigen capture ELISAs used the immuno-affinity purified antibodies (500 ng/well and 100 ng/well for S100A12 and PTX3, respectively) as capture antibodies and biotinylated forms of the same antibodies (1 μg/ml) for detection [69,70]. The biotinylated antibodies were detected with HRP-streptavidin-biotin complex (Amersham Life Science, UK) (1/500 in PBS). Milk samples were centrifuged (2,500 × *g*, 20 min, 4°C), the cream layer removed, recentrifuged (100,000 × *g*, 4°C, 30 min) and filtered (0.45 μm filter). Milk whey was diluted 1 in 5 with PBS for S100A12 and 1 in 2 for PTX3 detections and 100 μl added after coating the plate with the primary affinity purified antibody. The concentrations of S100A12 and PTX3 in milk whey were calculated from standard curves.

The S100A12-specific antigen capture ELISA was also used to measure the concentrations of S100A12 in milk whey samples obtained from 38 dairy cows (Corstorphine Dairy, Camden NSW, Australia). The milk samples were stored in 30 ml herd recording tubes, each pre-loaded with the milk preservative bromopol, and stored at 4°C. The somatic cells in the milk samples were measured using laser based flow cell cytometry with a Bentley Somacount 300 (Bentley Instruments Inc., Minnesota, USA) [71]. Samples were warmed to 40°C prior to SCC analysis to ensure all fat was mixed back in the sample.

### SDS-PAGE and immunoblots

The conditions used for SDS-PAGE and immunoblotting have been described elsewhere [72]. Unless specified, all samples were run under reducing conditions. Immuno-affinity purified antibodies to rS100A12 and rPTX3 were used in the immunoblots at final concentrations of 1 and 0.5 μg/ml, respectively. The same concentration of purified rabbit antibodies from pre-vaccination serum did not recognise either of the recombinant proteins in control immunoblots.

### Cloning, expression and purification of recombinant PTX3 expressed in insect cells

The cDNA encoding the mature PTX3 polypeptide was cloned into the insect cell expression vector pIEx5 (Novagen) (Table 5). The vector encodes the signal peptide of adipokinetic hormone thus potentially resulting in secretion of the recombinant protein into the culture medium. The plasmid was transformed into XL1-Blue cells and purified using an Endo-free Maxi plasmid kit (Qiagen). *Trichoplusia ni *insect cells (Protein Expression Facility, University of Queensland) were grown in serum-free Sf900-II^® ^medium (GIBCO) (1.1 × 10^6 ^cells/ml) and transiently transfected using 800 μl of Insect GeneJuice^® ^transfection reagent (Novagen) and 150 μg of endotoxin-free plasmid for each 100 ml of cells [73]. Cells were grown at 28°C (120 rpm; 48 h) at and the culture medium containing secreted recombinant PTX3 (rtnPTX3) was harvested (400 × *g*, 10 min). The medium was filtered, concentrated and dialysed into 20 mM Tris-HCl, pH 8.0, 1 mM EDTA, 5 mM benzamidine. The rtnPTX3 was then purified using anion exchange chromatography (Q-Sepharose).

### Reduction and alkylation of rtnPTX3

Purified rtnPTX3 or rPTX3 (each 100 μg in 100 μl) were mixed with an equal volume of 8 M urea in 20 mM Tris-HCl, pH 8.0 to denature the protein. Dithiothreitol (15 μl of 45 mM) was added and incubated at 50°C for 15 min. Freshly prepared iodoacetamide (16.5 μl of 100 mM) was subsequently added to the reduced samples which were incubated in the dark at RT for 45 min.

### Bacterial growth inhibition

Assays to measure inhibition of bacterial growth were performed using the Broth Micro-dilution Susceptibility Testing Method [45]. A frozen stationary culture of *E. coli *(LE392) was streaked onto LB agar plates and a single colony was subcultured in 6 ml of Muller-Hinton broth and incubated at 37°C (200 rpm, 5 h). Mid-logarithmic growth phase bacteria were diluted 1:250 in TBS, pH 7.2. Two fold serial dilutions of rS100A12 and the controls (antibiotics or BSA) were prepared in sterile polypropylene plates (Nunc, Roskilde, Denmark) compatible with cationic peptides. Purified rS100A12 (50 μg/ml), control antibiotics (25 μg/ml ampicillin and 12.5 μg/ml kanamycin) and BSA (80 μg/ml) in TBS pH 7.2 were used in the assays. Each plate also included wells containing the bacterial inoculum without any additives. Plates containing 10 μl of the diluted test compounds, 100 μl of 2× Muller-Hinton broth and 80 μl of water were then inoculated with 10 μl of diluted cultures (1 × 10^5 ^cfu/ml) in a final volume of 200 μl of 1× strength Muller-Hinton broth and incubated for 24 h. The plates were examined after 16 h and bacterial cell growth assessed by measuring the optical density at 620 nm (Spectramax Plus microplate reader, Molecular Devices, Sunnyvale, CA, USA).

### Binding of rtnPTX3 to bacteria

Colonies of low pathogenicity strains of *S. aureus *(ATCC 25923) [74] or *E. coli *(ATCC 35218) were grown in 50 ml Falcon tubes containing 20 ml LB Broth (37°C, 16 h, 200 rpm). The bacterial cultures were pelleted (3,000 × *g*), washed in PBS, pH 8.0 and diluted to an OD^600 nm ^of 0.8 (2.5 × 10^9 ^bacteria/ml). 25 ml of each bacterial sample was subsequently mixed with 25 mg of Sulfo-NHS-LC-Biotin (Pierce) according to the manufacturer's recommendations. Unbound biotin was removed by 3 washes with PBS and the pellets resuspended in 25 ml of PBS, pH 8.0. rtnPTX3 (50 μg) was immobilised onto HyBond C nitrocellulose (GE Healthcare Life Sciences) using a Bio-Dot^® ^SF Microfiltration Apparatus (BioRad). The negative controls were PBS or BSA. The membranes were then blocked using 5% BSA in TBS (0.05% Tween), washed and incubated overnight with 2 × 10^9 ^biotinylated bacteria (in 25 ml) with gentle rocking at 4°C. The membranes were then washed and incubated with HRP-Streptavidin-Biotin complex (1:500) in TBS for 2 h (RT), followed by three washes with TBS. Colour was developed using enhanced chemiluminescence (Pierce ECL Western Blotting Substrate).

### Binding of rtnPTX3 to human C1q

Binding of rtnPTX3 to human C1q was examined as previously described [43,46]. Briefly, 96-well plates were coated with 500 ng of human C1q (Merck) in 100 μl of PBS, pH 7.4 with 1 mM CaCl_2 _and 1 mM MgCl_2 _(4°C, ON). Wells were washed with PBS plus 0.05% Tween 20, blocked with 0.5% milk powder in PBS-Tween 20 (2 h at RT) before addition of 100 μl of different preparations of rtnPTX3 (each 12.5 ng) in PBS-Tween 20 (1 h at 37°C). The wells were washed in PBS-Tween 20 and incubated with 1 μg/ml of the immunoaffinity purified rabbit antibody raised to rPTX3 (1 h at 37°C), washed again and incubated with 1 μg/ml of HRP-conjugated anti-rabbit IgG (1 h at 37°C). After further washing, 200 μl of 2,2'-azino-bis (3- ethylbenzthiazoline-6-sulfonic acid) was added for 20 min. The reaction was stopped by addition of 50 μl of 4% SDS and absorbance at 405 nm measured.

## Authors' contributions

CPG and TV were responsible for the experimental *S. aureus *challenge of cows and sample collection. PAS aided the identification of cows and collection of samples while RW assisted with the design of the challenge experiment. AR performed the statistical analyses of the microarray data. LD performed the microarray analysis while YCSL, TV and CPG undertook the qRT-PCR analyses. LD expressed rS100A12 and YCSL expressed rPTX3 in bacteria. YCSL performed the S100A12 and PTX3 immunoblots and the PTX3 ELISAs. YCSL, KAB and LD carried out the S100A12 ELISAs. KAB performed the anti-bacterial assays with rS100A12. YCSL expressed and purified rtnPTX3 in insect cells. RDP assisted with the purification of recombinant proteins and polyclonal antibodies and performed the N-terminal sequencing of the recombinant proteins. RLT conceived the study, and participated in its design, coordination, analysis and manuscript writing. All authors read and approved the final manuscript
